# Synthesis and Antifungal Activity of Some Organotin(IV) Carboxylates

**DOI:** 10.1155/MBD.1998.233

**Published:** 1998

**Authors:** Josiah J. Bonire, G. Adefikayo Ayoko, Philip F. Olurinola, Joseph O. Ehinmidu, Neelam S. N. Jalil, Andrew A. Omachi

**Affiliations:** 1 Department of Chemistry Ahmadu Bello University Zaria Nigeria; 2 Department of Chemistry University of Queensland Brisbane QLD 4072 Australia; 3 Depatment of Pharmaceutics and Pharmaceutical Microbiology Ahmadu Bello University Zaria Nigeria

## Abstract

Six diorganotin(IV) carboxylates prepared by reacting diorganotin(IV) dichlorides with the respective silver carboxylate have been tested for antifungal activity against *Aspergillus*. *niger*, *Aspergilluus flavus* and *Pencillium*. *citrinum* in Sabourand dextrose broth. The compounds generally exhibit greater fungitoxicity than the diorganotin(IV) dichlorides and the carboxylic acids from which they were synthesized. In keeping with the generally accepted notion that the organotin moiety plays an important role in deciding the antifungal activity of an organotin compound, the diphenyltin(IV) compounds were more active than their di-n-butyltin(IV) analogues. However, the order of increasing fungitoxicity of the compounds parallels that of the uncomplexed carboxylic acids. The implications of the results are discussed.


### SYNTHESIS AND ANTIFUNGAL ACTIVITY OF SOME ORGANOTIN(IV) CARBOXYLATES

Josiah J. Bonire[1], G. Adefikayo Ayoko*[2], Philip F. Olurinola[3], Joseph O.Ehinmidu[3],Neelam. S. N. Jalil[1], and Andrew A. Omachi[1]

[1]Department of Chemistry, Ahmadu Bello University , Zaria, Nigeria.

[2] Department of Chemistry, University of Queensland, Brisbane QLD 4072, Australia.

[3] Department of Pharmaceutics and Pharmaceutical Microbiology, Ahmadu Bello University, Zaria, Nigeria

**Abstract**
Six diorganotin(IV) carboxylates prepared by reacting diorganotin(IV) dichlorides with the respective silver carboxylate have been tested for antifungal activity against *Aspergillus. niger, Aspergilluus flavus* and *Pencillium. citrinum* in Sabourand dextrose broth. The compounds generally exhibit greater fungitoxicity than the diorganotin(IV) dichlorides and the carboxylic acids from which they were synthesized. In keeping with the generally accepted notion that the organotin moiety plays an important role in deciding the antifungal activity of an organotin compound, the diphenyltin(IV) compounds were more active than their di-n-butyltin(IV) analogues. However, the order of increasing fungitoxicity of the compounds parallels that of the uncomplexed carboxylic acids. The implications of the results are discussed.

### Introduction

The chemistry of tin compounds continues to be of interest on account of their interesting structural[1] features and also because of their potentials as agricultural biocides[2-5], antitumor agents[6,7], wood perservatives[8], antioxidants for polypropylene[9], stabilizers for polyvinylchloride[10], marine antifouling coatings[11], antiherpes agents[12], flame retardants and and smoke suppressants[13], antiwear agents[14] homogenous catalysts[15] and recycling agents[16]. Previous investigations of the coordinating properties of carboxylates towards organotin compounds[3-6, 17, 18] have led to the isolation of new organotin(IV) carboxylates with antimicrobial or antitumor activity. On the other hand, the antifungal properties of salicylic acid and its derivatives have long been recognised[19]. We now report the antifungal properties of some salicylic, acetylsalicylic, acetic and phthalic derivatives of organotin(IV) complexes in the search for fungicides.

### Materials and Methods
All reagents were of reagent grade. Dibutyltin dichloride, diphenyltin dichloride, silver oxide and the carboxylic acid were obtained from Aldrich Chemical Company. The diorganotin dichlorides were recrystallized from $CH_2Cl_2$/hexane before use. Infrared spectra (as KBr discs) were recorded using Perkin-Elmer PE-700 and Perkin-Elmer Fourier Transform 1710 spectrometers. Elemental analyses (C and H) were carried out at the University of Sussex, UK while tin was determined as described by Farnworth and Pekola[20].

Procedures for the preparation of the organotin(IV) carboxylates used in this study are available in the literature[17, 21-25]. We, however, prepared the compounds using a new procedure, which employs the reaction of the relevant silver carboxylate with di-n-butyltin dichloride or diphenytin dichloride. The silver salt was usually prepared by the reaction of the carboxylic acid with one mole equivalent of silver oxide in $CH_2Cl_2$ and used as soon as possible after preparation. In a typical reaction, $Bu_2SnCl_2$ (4.6g, 0.015mol) in $CH_2Cl_2$ (150 $cm^3$) was stirred in the dark with $AgO_2CCH_3$ (6.50g, 0.039mol) for 24h and then filtered. The filterate was evaporated to dryness and the residue dried *in vacuo*. The crude product was purified by recystallization in toluene to give samples suitable for spectroscopic and elemental analyses.
A similar procedure was followed for the preparation of the other diorganotin dicarboxylates. However, 1:1 (silver pthalate: organotin dichloride) mole ratios were used for the preparation of the organotin phthlates.

The antifungal activities of the compounds were evaluated in Sabouraud dextrose broth by a method essentially similar to that described by Gershon and his co-workers[26]. However, in place of dimethyl sulfoxide used in that study, acetone was employed to dissolve the toxicants in the present investigation. Minimum Inhibiting Concentrations (MICs) of the compounds against *A. niger*, *A. flavus* and *P. citrinum* were obatined in $\mu gmL^{-1}$ by serial dilution of their acetone solutions and recalculated to a molar basis for comparison.

**Results**

The reactions of the silver carboxylates and diorganotin(IV) dichlorides afforded comparatively higher yeilds (78-90%) of the diorganotin carboxylates than those reported in literature[17, 21-25]. Elemental analyses (Table 1) suggested that the compounds were formed by simple substitution of the chloride ions by the carboxylate groups in keeping with the overall reactions below.

$R_2SnCl_2 + 2AgOCOR' \rightarrow R_2Sn(OCOR')_2 + 2AgCl$ (1)

$^1H$ NMR data were similar to those described in the literature[7, 21-25].

Table 1: Yield and Analytical Data of the compounds

| No | Compound | Yield (%) | Elemental analyses (calcd)% C | H | Sn |
|---|---|---|---|---|---|
| 1 | $Bu_2Sn(CH_3COO)_2$ | 90 | 41.09 (41.06) | 6.91 (6.81) | 33.49 (33.81) |
| 2 | $Bu_2Sn(2\text{-}HOC_6H_4COO)_2$ | 85.5 | 52.06. (52.10) | 5.52 (5.57) | 23.01 (23.40) |
| 3 | $Bu_2Sn(2\text{-}CH_3CO_2C_6H_4COO)_2$ | 90 | 52.98 (52.82) | 5.54 (5.46) | 19.65 (20.07) |
| 4 | $Bu_2Sn(1,2\text{-}C_6H_4(COO)_2)$ | 85 | 48.48 (48.37) | 5.72 (5.60) | 29.10 (29.89) |
| 5 | $Ph_2Sn(CH_3COO)_2$. | 80 | 49.13 (49.10) | 4.16 (4.09) | 29.90 (30.36) |
| 6 | $Ph_2Sn(1,2\text{-}C_6H_4(COO)_2)$ | 78 | 55.12 (54.92) | 3.36 (3.20) | 26.30 (27.16) |

The infrared spectra of the compounds were recorded in the range 600-4000 $cm^{-1}$ and the results are presented in Table 2. The strong asymmetric stretching bands of the carboxylates occur at *ca.* 1400 $cm^{-1}$ and the symmetric stretch at *ca.* 1550 $cm^{-1}$, confirming the success of the substitution reactions. The Sn-Ph deformation bands were at 1050 $cm^{-1}$, Sn-O-C at *ca.* 980 $cm^{-1}$ and Sn-C(alkyl) between 600 and 700 $cm^{-1}$

Table 2 : Important Infrared Bands of the Complexes ($cm^{-1}$)

| Compound | $\nu(CO_2)$ asym stretch | $\nu$(C=O)sym stretch | $\nu$(aromatic C-H stretch) | $\nu$(Sn-Ph) | $\nu$(SnOC) stretch | $\nu$Sn-Bu rock |
|---|---|---|---|---|---|---|
| 1 | 1560s | 1420s | - | - | 980m | 660w |
| 2 | 1580m | 1420m | 3060w | - | 995w | 640w |
| 3 | 1590m | 1430m | 3095w | - | 930w | 700w |
| 4 | 1560m | 1425m | - | - | 1000w | 680w |
| 5 | 1520m | 1390m | 3020w | 1055w | 980w | - |
| 6 | 1540m | 1435m | 3060vw | 1050w | 100w | - |

s=strong ; w=weak; m=medium; vw= very weak.

The antifungal activity of these compounds and of the organotin compounds and the carboxylic acids from which they were prepared is reported in Table 3. It is evident from Table 3 that the parent compounds viz. n-$Bu_2SnCl_2$ and $Ph_2SnCl_2$, phthalic acid, salicylic acid, acetic acid and acetylsalicylic acid as well as the individually screened derivatives exhibit varying degrees of fungicidal activity against the fungi used. A close look at the table reveals some instructive details. Firstly, the organotin carboxylates have lower MICs than the organotin chlorides and carboxylic acids from which they were derived. Secondly, the di-n-butyl compounds are usually more effective against the fungi than their diphenyl analogues. Thirdly, the most potent antifungal organotin compound in the series is **5** and the least is n-$Bu_2SnCl_2$ ie the order is **5**>**6**>$Ph_2SnCl_2$>

[JCO]2

**3**>**2**> **1**>**4**>n-$Bu_2SnCl_2$. Amongst the carboxylic acids tested, the order of antifungal activity is acetylsalicylic acid> phthalic acid> acetic acid> salicylic acid.

The MICs also show that the activity of the di-n-butyltin dichloride is increased 16.4, 3.3, 2.3, and 1.3 times on substituting acetylsalicylate, salicylate, acetate and phthalate groups respectively for the chloride group in the parent organotin chloride. Similarly, the antifungal activity of the diphenyltin dichloride is enhanced 5.0 and 1.3 times by substituting the acetate and phthalate groups respectively for the chloride groups in the starting material.

Table 3: Minimum Inhibition Concentrations (MICs) (in mmol/L) of the compounds against fungi species.

| Compound | *A. flavus* | *A. niger* | *P. citrinum* |
|---|---|---|---|
| **1** | 7.12 | 7.53 | 7.44 |
| **2** | 4.93 | 4.98 | 5.19 |
| **3** | 0.87 | 0.86 | 0.84 |
| **4** | 12.59 | 12.59 | 12.59 |
| **5** | 0.13 | >0.13 | >0.13 |
| **6** | 0.57 | 0.57 | 0.57 |
| $Ph_2SnCl_2$ | 0.73 | 0.72 | 0.77 |
| $Bu_2SnCl_2$ | 16.45 | 8.22 | 16.45 |
| Salicylic acid | 18.02 | 18.10 | 18.09 |
| Acetic acid | 16.38 | 16.38 | 16.38 |
| Phthalic acid | 15.04 | 15.05 | 15.05 |
| Acetylsalicylic acid | 13.87 | 15.05 | 15.04 |

### Discussion

Although the antifungal activity of salicylic acid derivatives have long been known[19], relative to the organotin compounds used in the current work, the free carboxylic acids presented weak antifungal activity against the tested fungi. We define good activity as inhibition below 0.1 mmol/L; moderate activity as 0.1-1.0 mmol/L and weak activity as greater than 1.0 mmol/L This indicates that the presence of the metal ions plays an important role in the increased antifungal activity when these acids are coordinated. In this respect, our results are consistent with a well-known fact that many[27, 28] biologically active compounds become more active upon complexation than in their uncomplexed forms.

On the other, the fact that the organotin carboxylates are more active against the tested fungi than their parent organotin chlorides suggests that the carboxylate groups play a role in the fungitoxicities of these compounds. This seems particularly true for the di-n-butyltin carboxylates since the order of fungitoxicites of the free acids roughly parallels that of the carboxylates. According to Crowe[29], the actual biological activity of diorganotin compounds of the type RR′SnXY is determined solely by the $RR'Sn^{2+}$ moiety. Consequently the group XY would only influence the delivery of the active $RR'Sn^{2+}$ ion to the cell. However, since the free carboxylic acids show some antifungal activity, it seems unlikely that the activity of the $RR'Sn^{2+}$ moiety only provides full explanation for our results. The higher activities of the carboxylates relative to their parent organotin compounds and the free carboxylic acids appears to be an additive (not a synergistic) effect of the metal ions and the carboxylate groups, with the possibility of a common mode of action. In this regard, the hypothesis[28] that relates the toxicity and nontoxicity of metal complexes to the penetration and nonpenetration of the fungus by the toxicant is of interest. Groups like the acetylsalicylate and $RR'Sn^{2+}$ considerably change the molecular parameters that influence the orientation of the molecule on biological receptors and facilitate penetration into the cell membranes.

The observation that the diphenyl compounds are more active than their di-n-butyl analogues is in line with the notion that the number of carbon atoms in the organotin moiety [29] affects its activity. In the triorganotin series, optimal activity has been associated with nine to twelve carbon atoms [30] and this may be the case in the present work, where the diphenyltin group contains twelve carbon atoms but their di-n-butyltin- counterparts contain eight. However, it is noteworthy that Gielen and his co-workers [6] found that some di-n-butyltin(IV) derivatives of salicylic acid were more active than the corresponding diphenyl- or diethyl-compounds.

Our results clearly indicate that the organotin(IV) carboxylates described in this work show antifungal activity. Further investigations of these and related compounds with different fungi are in progress.

**References**

1. E. R. T. Tiekink, *App. Organomet. Chem.* **5**, 1 (1991).
2. A. J. Crowe, *App. Organomet. Chem.* **2**, 143 (1987).
3. J. J. Bonire, P.F. Olurinola, M.A. Ibrahim and J.O.Ehinmidu, *Proc. Natl. Sci. Conference of National Association of Academic Pharmacists,* 105 (1992).
4. P.J.Smith and A.G. Davies *Intern. Tin Res. Publ.* Pub. No 618, p510 (1987).
5. P.J.Smith and L. Smith, *Chem. Br.*, **11**, 208 (1975).
6. M. Gielen, P. Lelieveld, D. de Vos and R. Willem in B. Keppler (ed), *Metal Complexes in Cancer Chemotherapy*, VCH Weinheim, p383 (1993).
7. M. Gielen, *Coord. Chem. Rev.* **151**, 41, (1996) and references therein.
8. S. J. Blunden and R. Hill, *App. Organomet. Chem.*, **4**, 63 (1990).
9. M. Bevilacqua, M. Pereyre and B. Mailalard, *App. Organomet. Chem.*, **10**, 477 (1996).
10. S. Karper, *Tin and its Uses*, No 162, 6 (1990).
11. S. J. Blunden and R. Hill, in *"Surface Coatings-1"*, A. D. Wilson, J. W. Nicholson and H. J. Prosser (ed), Elsevier Applied Science Publishers, pp17-67 (1987).
12. S. G. Ward, R. C. Taylor and A. G. Davies, *App. Organomet. Chem.* **2**, 47 (1988).
13. R. S. Bains, P. A. Cusack and A. W. Monk, *Eur. Polymer J.* **26**, 1221 (1990).
14. S. Karpel, *Tin and its Uses*, No 157, 12 (1988).
15. S. J. Blunden, P. A. Cusack and R. Hill, in "*The Industrial uses of tin chemicals*", The Royal Society of Chemistry, London. (1985).
16. F. Vohwinkel, *Tin and its Uses*, No 149, 7 (1986).
17. A. Mariem, R. Willem, M. Biesemans, B. Mahieu, D. de Vos, P. Lelieveld, M. Gielen, *App. Organomet. Chem.* **5**, 195 (1991).
18. J. J. Bonire, *Polyhedron*, **4**, 1707 (1985).
19. L. V. Coates, D. J. Brain, K. H. Kerridge, F. J. Marcus, and K. Tattershall, *J. Pharm. Pharmacol.,* **9**, 855 (1957).
20. M. Farnworth and N. J. Pekola, *Anal. Chem.* **31**, 410 (1961).
21. D. L. Alleston and A. G. Davies, *J. Chem. Soc.,* 2050 (1962).
22. R. D. Dworkin and A. J. Ejk, *M and T Chemicals Inc Ger. Offen.*, 262654, (1975/6); *C. A.* **86**, 140253 (1977).
23. D. V. Nauk, J. C. May, C. Curran, *J. Coord. Chem.* **2**, 309 (1973).
24. D. P. Graddon and B. A. Rana, *J. Organomet. Chem.*, **136**, 19 (1977).
25. Y. Maeda, and R. Okawara, *J. Organomet. Chem.* **10**, 247 (1967).
26. H. Gershon, D.D. Clarke, and M. Gershon, *J. Pharm. Sci.* **80**, 542 (1991)
27. F. Caruso, M. Bol-Schoenmakers and A. H. Penninks, *J. Med. Chem.* **36**, 1168, (1993) and references therein.
28. H. Gershon, *J. Med. Chem.*, **17**, 824 (1974).
29. A. J. Crowe, in *Metal-based Antitumor Drugs*; M .Gielen (ed), Tel Aviv, Freund Publishing House, **1**, 103 (1989).
30. Z. H. Chohan and A. Rauf, *Synth. React. Inorg. Met-Org. Chem.* **26**, 591 (1996) and references.

**Received: July 3, 1998 - Accepted: July 14, 1998**

